# Exposing the profiteers of globalisation, disaster, conflict and detention

**DOI:** 10.4102/jamba.v8i1.376

**Published:** 2016-10-31

**Authors:** Jason K. von Meding

**Affiliations:** 1 School of Architecture and Built Environment, University of Newcastle, Australia

In *Disaster capitalism: Making a killing out of catastrophe*, acclaimed Australian journalist Antony Loewenstein turns his passion for justice to deliver a stunning critique of the thriving disaster capitalism industry, in its many forms; the profiteers of privatised detention, militarised security, the aid industry and multinational mining are relentlessly skewered with style and poise, and their predatory tactics exposed.

*Disaster capitalism* is the story of Loewenstein’s journey into the belly of this particular beast. The book gives us an up-close and personal look at how corporations like Serco, G4S, Halliburton and their ilk profit from organised misery, perpetual conflict and the impacts of disaster, and how national governments and international organisations like the IMF and the World Bank are willing collaborators. In Part I, he takes us to Afghanistan, Pakistan, Papua New Guinea and Greece, exposing the various exploitative strategies employed to enrich the local elite and foreign interests, and the devastating effects on the majority of people in each country. In Part II, we visit wealthy Western democracies (Australia, the United States and the United Kingdom) that punish the most vulnerable in their societies while dictating economic conditions to the world, imposing taxpayer funded cruelty for private profit at home and abroad.

This is an absolutely enthralling read, a must for the revolutionary, the dreamer, the activist, the teacher and the learner. Loewenstein has compiled a treasure trove of evidence on his travels. His dismantling of the social and economic myths that enable predatory disaster capitalism is robust and compels us to action. He offers a ‘challenge to cherished beliefs concerning aid and development, war and democracy, and in particular the modern, borderless nature of capitalism’ (p. 14). For this reader, three key themes emerge: a dialogue around crime and punishment; a critique of the idea of benevolent corporations; and the grim reality that this is all part of a plan, a rigged system that empowers and enables predator capitalists to flourish.

## Crime and punishment

As the prison industrial complex has rapidly taken hold in Western societies, the public clearly favours an ideology of punishment over reform. In addition to highlighting issues around race and class, Loewenstein speaks to issues around the treatment of those in the care of the state, and how ‘lobbying, ideology and a punishment ethos have colluded to produce one of the most destructive experiments in recent times: mass incarceration’.

Judicial processes in the United Kingdom, United States and Australia target the marginalised for what amounts to, essentially, punishment for being unable to escape their systemic disadvantage. Loewenstein unpacks the ideology behind this phenomenon and asks whether the poor man, the petty criminal, the asylum seeker or the drug user really deserve the punishments prescribed, and who indeed benefits? What of the bankers that caused a global financial collapse? The CEOs of corporations that destroy the only planet we have? The heads of state who lied in order to enable the invasion and destruction of Iraq, leading to the destabilisation of the region and a current displacement crisis of epic proportions? Should not our justice system be designed to protect society from such individuals and the devastating consequences of their actions?

From June 2016, we have witnessed a brutal crackdown on drug sellers and users in the Philippines, since the rise to power of President Duerte. Summary executions on the streets have shocked the world, yet few official condemnations are forthcoming (apart from regarding his lack of diplomacy). Although it is not difficult to imagine that many politicians and indeed members of the public might secretly support these abuses of power and share the President’s disdain for Article 10 of the Declaration of Human Rights, as Loewenstein finds in Australia, United States and United Kingdom, there is an infinitely more ‘subtle’ way to enforce the harshest punishments: through private contractors.

The criminal justice system in Australia ensures sky-high rates of Aboriginal incarceration, and, as the recently revealed abuses of the NT government demonstrate, the hateful punishment of those discarded by society is absolutely state-sanctioned. In America, the black population is also disproportionately incarcerated. Loewenstein explores the roots of a system that enables this in the United States and the corporations that profit handsomely at the expense of taxpayers, destroying families and leaving little opportunity for rehabilitation and reintegration into society. ‘Private prison corporations saw a unique opportunity’ (p. 196) in America, Loewenstein writes, to do everything possible to ensure that more and more people were incarcerated. The prison population is 30 times what it was in the 1990s. The absolutely failed ‘War on Drugs’ has wreaked havoc on society. For all the posturing about market efficiency, private prison corporations are a spectacular leech off the government purse, with a rigged legal system providing financial and political benefits right down the food chain. All of this is possible, he tells us, because of a lack of ‘serious questioning of the harsh, punitive ideology underpinning US “justice”’ (p. 207).

In Australia, United Kingdom, United States and Greece, Loewenstein exposes the fact that asylum seekers and migrants are also punished, most often without breaking any law. In Greece, he provides a rich cultural background of ‘not just economic harshness, but a culture that tolerated and celebrated exclusion’ (p. 69). In the grips of imposed austerity measures, the social fabric began to unravel and ‘Popular frustration was taken out on the most marginalized group in society: refugees’ (p. 72). The mandate for demonisation of the vulnerable that was secured in Greece, as in Australia, was just one tactic used to ensure profit for human rights abuses across the countries that Loewenstein investigates.

Time and again, Loewenstein finds governments all too eager to enable those corporations in a position to cash in. He details how the European Union (EU) has become central in ‘funding, encouraging and pressuring EU nations to isolate and imprison asylum seekers’. He discusses the industries that have sprung up and thrived, often with the EU leading ‘the charge in working with corporations that have been very willing to develop and hone methods for repelling the desperate hordes’. As ‘Fortress Europe’ closes her borders, deals like that done between the EU and Turkey are sealed without a second thought for the human cost. Corporations and corrupt governments profit; the vulnerable are turned away and suffer.

## Benevolent corporations

Loewenstein picks up where Naomi Klein left off in her 2007 bestseller *Shock Doctrine*. She pointed out that privatisation of government has accelerated in the United States, as private sector opportunities have been generated through the ‘war on terror’. She argues that:

now wars and disasters are so fully privatized, that they are themselves the new market: there is no need to wait until after the war for the boom – the medium is the message. (p. 13)

Loewenstein builds on this and adds that:

it is hard to escape the conclusion that wars are often fought for the key reason of liberating new and willing markets – and with the war on terror likely to continue for decades, there will be no shortage of new business to secure. (p. 16)

We often encounter the myth of the benevolent corporation. As much as it might be comforting to believe that the private sector simply goes about its business in a free market generating jobs and growth, from cover to cover *Disaster capitalism* lays bare the impacts of a global privatisation bonanza. For Loewenstein, the United States has played a pivotal role. He says that a ‘central plank’ of US foreign policy is ‘the US model of reducing the role of government while increasing the influence of largely private power has never been so rapacious, though the problem is global’ (p. 4).

Loewenstein is no admirer of market fundamentalism, saying that ‘wealth is concentrated in so few hands in today’s world: there is little incentive to advocate for a more equitable planet. The market system guarantees unfairness and rewards greed’ (p. 2). He shows us examples of open rebellion against this system from communities in Greece, Haiti and PNG, countries exploited long and hard by the status quo. As we have become more enslaved to the neoliberal project, Loewenstein argues ‘that the corporation is now more powerful than the nation-state, and that it is often the former that dictates terms to the latter’ (p. 7).

In Bougainville, PNG, Loewenstein meets members of the resistance against resource exploitation and explores the shady relationships between corporate and political interests. The memories of violence fuelled by greed and repression do not fade easily. The health of the community and the environment has also been terribly compromised. ‘Environmental vandalism should not be the price tag for “progress”’, he pleads.

In Afghanistan, we are introduced to Jack, the British MD of a private military company (PMC) who provides an inside look at a truly burgeoning industry. He is not shy to admit that his corporation ‘survives off chaos’ (p. 20). Jack anticipates perpetual war and opportunity. ‘If we can make money, we’ll go there’, he tells Loewenstein. He sees his industry in a purely positive light, providing ‘jobs for the boys leaving the army who can continue their trade’. In spite of the well-documented abuses of PMCs in Afghanistan and Iraq, military objectives continue to be dressed in humanitarian robes, government intelligence gathering has been privatised and mercenaries are ensured ‘a quick buck’ (p. 21). Indeed, Loewenstein finds that the PMC industry hopes that the conflict and the profit will never end. When it does, they will be ‘looking for the new war’ (p. 61).

How often are we outraged at government spending on weaponry and conflicts that we deem unnecessary, but hesitate to question the relationship between corporate interests and government policy and spending. Loewenstein reminds us that the war on terror represents one of the largest wealth transfers in history, with 4 trillion dollars to date being spent, with much of it going to ever-grateful Western contractors. The privatisation of prisons and security apparatus is incredibly expensive, while all evidence shows that incarceration does not tackle societal problems that lead to crime, but rather reinforces them.

The overwhelming message is that simply outsourcing your cruelty is a convenient way to avoid responsibility, transparency and accountability, while profiting corporations and manipulating the economy. Neoliberal governments would like us to accept the notion that corporations are ultimately benevolent entities that exist only to employ people, satisfy market demand and grow GDP. Loewenstein argues that ‘multinational corporations spent the twentieth century gradually reducing their obligations in the various jurisdictions in which they operated’ (p. 243). What we have now is unregulated, unaccountable and secretive private sector entities. Meanwhile, governments with dirty work to outsource are not left disappointed.

Unfortunately, a wilful ignorance of the sometimes devastating social impact of ‘business’ has allowed a mentality of self-righteousness to fester, completely detached from the suffering of people that stand in the way of profit, those targeted by governments for suppression and oppression, and the unfortunate citizens of countries outside of the US circle of trust, whose lives appear to hold so much less value than those of allies. Companies like DynCorp and Blackwater, despite having their abuses repeatedly exposed, thrive in this context.

## A rigged system

Loewenstein exposes, time and again, the fact that the global economy is dominated by anti-democratic and predatory forces that profit the wealthy and the ruthless. The revolving door between corporations, lobby groups and government is clear for all to see. This collusion between powerful actors fans the flames of crisis, while selling market fundamentalism as the antidote and positioning ‘benevolent’ corporations to reap the benefits. In the United States, the banks were bailed out, while personal debt and, indeed, poverty rates soar. Loewenstein offers a stinging critique of a system rigged for the 1% and the scandalous truth that in the United States, both major parties represent similar corporate interests, while the media feigns ignorance. Indeed, liberal presidents have done little for the vulnerable other than make empty promises.

Meanwhile, in Haiti, Loewenstein describes an environment of ‘canny capitalists sifting through the ashes of a disaster, looking for business opportunities’ (p. 109). His narrative of this historically vulnerable nation describes the strong 20th century American support for successive brutal dictatorships, enriching US interests and a local elite. We see this model replicated again and again in *Disaster capitalism* and indeed around the world as a key element of US foreign policy. The example, in Chapter 3, of the ‘devoutly anti-Communist’ ‘Baby Doc’ Duvalier is particularly damning, who ‘unlike the many African despots targeted by the Hague, remained a friend of the West and was therefore largely untouchable’ (p. 110). When the neoliberal agenda was challenged in Haiti by Aristade, the United States and local elite conspired to overthrow the government to restore ‘order’.

We are often presented with the assertion that the international community, led by US humanitarianism, rescued Haiti after the 2010 earthquake. Loewenstein paints a very different picture and claims that ‘when Haiti had received lashings of ‘help’ this generosity had done little but enrich foreign companies’ (p. 115). The local reception to UN intervention was largely hostile. In the context of historical US interventions in Haiti, this comes as no surprise, and the sentiment is well founded. As revealed by Wikileaks, the US ambassador to Haiti asserted that the UN military-style solution was ‘an indispensable tool in realizing core [*US government*] policy interests in Haiti’ (p. 115).

In a similar vein, most development aid to PNG from Australia since its independence either found its way into the pockets of either the wealthy PNG elite or Australian corporations. Far from its claimed humanitarian ideals, Loewenstein says that the main goal of the Australian government in PNG was simply ‘to ensure that Australian corporations had a ready market in which to turn a profit’ (p. 172). The denial of complicity with oppressors in the violent struggles of the 1980s and the patronising attitudes displayed by Australian diplomats leave a bitter taste.

Loewenstein reserves some of his harshest criticism for the mainstream media, and the ‘false construct of “balance” that permeates the corporate press, which merely pits one powerful interest group against another’ and one that ‘views business and political leaders as far more important than the individuals and societies affected by them’ (p. 10). As an independent journalist that opposes the state of his profession, he laments the fact that ‘90% of Americans rely on information from media outlets owned by only six multinationals, including News Corporation, Comcast and Viacom’.

## Conclusion

*Disaster capitalism* pulls no punches in calling out both profiteers and enablers. Loewenstein exposes a shady cabal operating in plain sight, corporations that will not blink at the thought of misery, death and destruction as part of business as usual. Governments that outsource their most distasteful projects to companies that have neither conscience nor boundaries will not be pleased with his outspoken style. They thrive on a complete lack of transparency and accountability that allows ongoing abuse to yield few consequences for the perpetrators.

The book is impossible to put down and rich with memorable lines. It will have the reader coming back to review the stories of friend and foe, of oppressed and oppressor. Loewenstein has skillfully articulated opposing positions, admitting his ideological bent where possible in the text and to those he meets in the field. It is sure to be a book both loved and hated, depending on the beliefs of the reader, for its honest storytelling. The accounts of his journalistic interactions give the book a very personal feel.

Loewenstein shows us how accepting something terrible (e.g. abuse of asylum seekers and mass incarceration) out of a fear of personal harm, insecurity or loss gives a perceived legitimacy to profiteers. He wrote the book to ‘shock, provoke and reveal’ (p. 16). The question is, once we know all about the profiteers of calamity, will we just carry on or will we fight for justice?

